# Communicative and Social Skills among Medical Students in Spain: A Descriptive Analysis

**DOI:** 10.3390/ijerph17041408

**Published:** 2020-02-21

**Authors:** Sonia Ruiz de Azua, Naiara Ozamiz-Etxebarria, Miren Agurtzane Ortiz-Jauregui, Ana Gonzalez-Pinto

**Affiliations:** 1 Department of Neuroscience, University of the Basque, Cibersam, 48940 Leioa, Spain; sonia.ruizdeazua@ehu.eus; 2 Department of Neuroscience, University of the Basque Country, 48940 Leioa, Spain; mirenagurtzane.ortiz@ehu.eus; 3 Alava University Hospital, Cibersam, University of the Basque Country, 01004 Vitoria, Spain; anapinto@telefonica.net

**Keywords:** empathy, active listening, risk communication, social skills, communication, medical student

## Abstract

Effective risk communication in public health requires the development of social skills such as active listening and empathy. Communicative and social skills were evaluated in third-year medical students (*n* = 917) using the Active Listening Test and the Test of Cognitive and Affective Empathy. The results obtained revealed that our participants had equal or better-than-norm communication skills. Women scored higher in active listening whereas men scored higher on the General Empathy Scale. The students who preferred a clinical specialty obtained higher scores in active listening and empathetic abilities, as compared to students who chose a surgical specialty. In conclusion, the medical students who participated in the study exhibited good communicative and social skills. An association was observed between gender and specialty preference, and empathy and active listening skills.

## 1. Introduction

The World Health Organization (WHO) defines risk communication as an essential component of risk analysis that is strictly linked to two other components: risk assessment and management, which are the basis of public health prevention strategies. The environmental impact of risk communication by public health institutions must be carefully planned and conducted on the basis of an accurate study of the context [1].

The emotional responses generated by these risks have several characteristics as a common denominator. Said characteristics have been called “outrage”. Being able to listen to these outrage factors is crucial in order to give an adequate response. Furthermore, in order to understand what kind of emotions are at play and what communicational approaches to use in order to improve people’s emotional responses in an emergency, it is important to have empathy [2].

Vincent T. Covello [3], when describing the seven cardinal risk communication rules, underlines the rule regarding the importance of listening to the public. It is vital to listen to people’s real concerns. Thus, it is crucial that doctors have the ability to listen actively. This is a social skill that demonstrates to the speaker that the listener has understood and does not make assumptions about what people know, think, or want. Active listening requires the participation of people, and our teammates and patients will appreciate a good active listening skill [4].

Another important skill during a crisis is an open and empathetic communication style. This communicative style engenders the public’s trust and is the most effective when officials are attempting to galvanize the population to take a positive action or refrain from a harmful act. Although trust is imperative in a crisis, the public’s distrust of scientific experts and government representatives are increasing for a variety of reasons, including access to more sources of conflicting information, a reduction in the use of scientific reasoning in decision making, and political infighting. Trust and credibility—which are demonstrated through empathy and caring, competence and expertise, honesty and openness, and dedication and commitment—are essential elements of persuasive communication [5]. In addition, felt empathy evokes cognitive and emotional processing that leads to important health-promoting responses [6,7,8,9,10].

Certain studies that analyze empathy in medical students show that there is a downward trend throughout the study of the degree of medicine. There are those who justify this trend as a method of protecting patients from suffering [11]. However, other studies contradict these results, as they conclude that empathy is maintained and even slightly increased in medical students [12].

A third factor that seems to have an impact on social skills is specialty. Those who prefer person-oriented specialties are more empathetic and sensitive than those who prefer surgical and technical specialties. Certain studies even conclude that it is possible to predict a student’s medical or surgical preference by evaluating his or her empathy [13]. In contrast, other studies have not documented any significant differences in the levels of empathy according to the specialty chosen [11]. Some authors credit these differences to the fact that women and students who prefer patient-centered specialties are more empathetic [12]. On the other hand, studies show that gender can be a determining factor in empathy among medical students.

Last, but not least, gender-based differences have been observed in social skills. A number of studies have shown that gender is a determining factor in the level of empathy, with women being statistically more empathetic than men [14]. Medical students are no exception; the literature states that women have more empathy from the beginning and lose less empathy during medical education as compared to male students [15].

The relationship between health education and risk perception in terms of environmental issues has not been studied in depth, and there is a limited number of studies on the subject [16]. These studies have suggested that in order to plan effective risk communication strategies, the levels of health literacy of target audiences must be taken into account [17]. The objective of this study was to evaluate the communicative and social skills that medical students needed in order to guarantee a good transmission of risk situations [3]. In this study, we evaluated the communication skills that constitute the basis of risk communication. However, risk communication is a highly complex process that requires more than basic social skills, as a physician has to be able to deliver messages, assume responsibilities, and provide solutions. We focused on the evaluation of empathy and active listening skills and performed a comparative study based on specialty preference and gender.

## 2. Materials and Methods

### 2.1. Participants

The sample was composed of 917 third-year students of the Faculty of Medicine of the University of the Basque Country, Bizkaia, Spain. Students were recruited from February 2019 to January 2020. Participation in the study was voluntary and participants were informed about the project’s procedure.

### 2.2. Instruments

Social skills and, more specifically, active listening and empathy were evaluated using two tests: Active Listening Test(ALT) and Cognitive and Affective Empathy Test; (TECA).

The Active Listening Test is composed of 20 items, with two response options (yes and no) and four scales: listening without interruption, listening paying 100% attention, listening beyond words, and listening encouraging the other to go deeper. The range of each scale was 0–5 points—0–2 scores show low abilities, 3–4 show normal abilities, and more than 4 indicate good active listening abilities.

The TECA test [18] was created to measure empathy. It consists of 33 items measured on a Likert five-point scale: (*“totally in disagreement”, “somewhat in disagreement”, “neutral”, “somewhat in agreement”*, and *“totally in agreement”*). This test consists of four evaluation scales, namely, adoption of perspectives, emotional understanding, the ability to identify negative emotional states (empathetic stress), and the ability to identify positive emotions (empathetic joy). There is a general scale of empathy. All direct scores must be standardized according to the tables recommended by the authors—0–6 scores are valued as extremely low, 7–30 scores are valued as low, 31–69 scores are valued as normal, 70–93 scores are valued as high, and 94–99 scores are valued as extremely high.

### 2.3. Procedure

This project was approved on February 20, 2019 by the Ethics Committee of the University of the Basque Country CEISH-UPV/EHU, BOPV 32, 17/2/2014, with code M10_2018_263. The research project was titled “Study on active listening capacity, empathy, assertiveness and social skills”.

The third-year medical school curriculum includes a subject titled “Communication and Clinical Relations” where students of our University are required to complete a battery of questionnaires. Through these questionnaires, sociodemographic data such as age, gender, marital status, number of siblings, and specialty preference were collected. In an initial stage, 213 students completed a hard-copy empathy test. Subsequently, we realized that the design of the study could be improved by collecting more sociodemographic data and recording specialty preferences. Thus, in a second stage, 704 medical students completed a battery of online questionnaires to assess their active listening skills and empathy, and collect more sociodemographic data such as civil status, parental status, and preferred specialty.

Students completed all questionnaires through Google forms using a code ensuring anonymity. At the end of the course, we gave the students feedback on the results of the tests. The estimated time to complete all of the tests was about 20 min.

### 2.4. Analysis

The statistical program SPSS 26 was used for data analysis. A database was built using the sociodemographic data and questionnaire results. Initially, the homogeneity of variables was tested using the Kolmogorov–Smirnov test. The non-parametric U Mann–Whitney statistic was used to assess differences in active listening and TECA scores according to gender and specialty.

## 3. Results

Of the 917 third-year medical students, 669 were women (73%) and 248 men (27%) (Table 1). Their ages ranged from 19 to 43 years, with an average of 20.86 years and a standard deviation of 2.19.

Third-year medical students scored higher than the normative median in “listen beyond the words”, “encourage to go deeper”, “adoption of perspectives”, and “emotional compression” factors. The other scores were in the normative mean.

There were significant gender-based differences in two subscales of the Active Listening Test, namely, “listening beyond words” and “encouraging to go deeper” factors, with women showing higher abilities than men. In contrast, no significant differences were observed in empathy scores. It was only in the General Empathy Scale that men had higher scores in empathy than women (Table 2).

Students were asked directly about their preferred medical specialty; some students were very certain about their preferences, whereas others still did not know what specialty to choose (168). All medical specialties were listed and classified into clinical, surgical, clinical-surgical, and laboratory categories. Most students chose clinical or surgical specialties, with fewer students preferring laboratory or clinical-surgical specialties. For this reason, statistical analysis was performed considering the most valued specialty. Table 3 shows how the students who chose a clinical specialty had higher scores in the “encouraging to go deeper” scale of Active Listening and in empathetic stress.

## 4. Discussion

In the Basque Country, more women are enrolled in college than men. In health sciences, the percentage of women is around 70% [19]. In our sample, young women accounted for 73% of our study population, with an average age of 20.86 years, which is consistent with the profile of medical students in Spain. The gender of participants could influence the results obtained.

In the Active Listening Test factors “listen beyond the words” and “encourage to go deeper”, participants scored higher than the norm. This was especially true for women, who had the ability not only to listen to the verbal message, but to non-verbal language as well. Female medical students were found to have better active listening skills, which is essential for risk communication [3]. In order to listen to the real concerns of the patient, it is necessary to go beyond the words and have deep-listening skills [3], which are more common among medical students than in the general population. For example, there are some aspects of communication in public health that, although they have a low incidence, have a high emotional impact [20]. This emotional impact can only be perceived if the physician has the ability to listen beyond the words. To summarize, it is important to understand and identify non-verbal messages conferred by the speaker’s tone and body language.

Regarding empathy, the participants of the study also obtained high scores in the adoption of perspectives and emotional comprehension. Our students have the ability to put themselves in another person’s shoes and understand the emotional state of others. On the other hand, their scores in empathetic stress or empathetic joy are within the norm. The emotional contagion for both positive and negative emotions is not higher in medical students than in the general population. Physician’s empathy benefits the patient, who shows increased adherence to treatment, greater satisfaction with the treatment, and fewer malpractice complaints. The benefits for the physician are increased health, well-being, and professional satisfaction [21], as well as decreased burnout, personal distress, depression, and anxiety [22]. A study found that high empathy is significantly associated with low burnout [23]. Therefore empathy has a protective role in burnout of primary care general practitioners (GPs) [24].

All these aspects of empathy demonstrate confidence and credibility when it comes to communication, and these elements are necessary for persuasive communication [5]. Emotional understanding and adoption of perspectives, factors in which participants obtained high scores, evoke the cognitive and emotional processing that leads to important health-promoting responses [7,8,9,10,25]. Empathy is essential to know what kind of emotions are at play and what types of communication should be used in risk communication [2].

There were no statistically significant differences between men and women in TECA scores. Men scored higher in empathy than women. This finding is inconsistent with cumulative evidence that there are no gender-based differences in terms of empathy [26,27,28], or that female medical students and physicians score higher in empathy than men [6,15,29,30,31,32]. Conversely, other research studies report higher scores for men in empathy [33]. This inconsistency can be explained by the low percentage of men included in our sample, as compared to other studies, and by the fact that men in our sample could be more emotionally attuned. Of note, the sample of male students was more heterogeneous in the study where men were found to be more empathic than women [33], and the results for men showed greater dispersion.

The students who prefer clinical specialties scored higher in “encourage to go deeper” and “empathetic stress factors” than those who preferred surgical specialties. The results of this study are consistent with multiple studies suggesting that students choosing people-oriented specialties (e.g., family medicine, pediatrics, oncology) scored significantly higher in comparison to students preferring technology-oriented specialties such as surgery or traumatology [12,31]. Perhaps, in the choice of specialty, students take an introspective look at their skills and choose specialties for which they have resources.

The medical curriculum requires acquiring a huge body of theoretical and technical knowledge, whereas psychological strategies are not a given priority. To be admitted to a Faculty of Medicine, students are required to have a high mark in the university entrance examination, which measures theoretical knowledge but not personal abilities, which are essential for an adequate medical practice. Communication skills may appear to be innate to the individual, but they vary from one person to another, and they also can be acquired. These skills, however, are not always required or acquired during medical education. Research suggests that communication skills do not necessarily improve with training or experience. Several studies show that empathy scores vary during undergraduate medical training. Thus, in some countries, empathy scores in clinical fields decreased [12,30,33], whereas empathy scores remained the same or even increased in other countries [34]. Taking into account that active listening and empathy are crucial for an effective physician–patient relationship, training empathy during undergraduate medical education is essential.

The University of the Basque Country offers a course called Ethics, Communication and Clinical Relationships. The aim of this subject is that students develop social and communication skills to be able to build a good doctor–patient relationship. Such training is expected to ensure that graduates not only acquire clinical but also emotional skills. Emotional skills help physicians understand what the patient is going through and make the patient feel more comfortable in a difficult situation. In this course, students acquire active listening skills, empathy, basic social skills, negotiation skills, clinical interviewing skills, bad-news communication skills, risk communication skills, and strategies to deal with complicated patients.

It is important to work on the communication skills of students to avoid increasing the outrage of the community, which is difficult to handle afterwards. Inappropriate communication is strongly related to the population’s perception of higher risk on low-incidence health issues [20]. In other words, good communication can contribute to the success of a risk management program [35].

The need to integrate communication skills in health science education has been recognized for years. Thus, this topic can be addressed both transversally and by including specific courses in degree programs. However, it is not always included in medical curricula, nor is it transversally worked on in clinical courses [36]. Communication skills should be taught in the Faculty of Medicine both, transversally and in specific courses. Communication skill training should also be offered in ongoing professional education. However, despite the supporting evidence and the recommendation to include them in the curriculum, social skills are often not taken into account in professional evaluation, and scientific knowledge is given priority [13].

Additional studies are needed to elucidate the impact of social and communicative training on the social abilities and risk communication skills of physicians. It is crucial that physicians develop relevant abilities such as being able to listen actively and being empathetic. It is also important that students participate in simulations of risk communication situations. Risk communication is a very complex process, especially when health is at stake. It is necessary that students learn to understand scientific information, are aware of and manage complexity, determine the reliability of different sources of information (fake news), manage conflicts of interest, deliver information in a clear manner, explain scientific concepts, and combine these factors with the information received by patients [37]. This is one of the avenues for future research that will be pursued in the University of the Basque Country.

## 5. Conclusions

This research revealed that the medical students who participated in this study have empathy and active listening skills. Physicians should have the same or better risk communication skills than the normative average.

As far as gender is concerned, women who participated in this study demonstrated a higher capacity for active listening than men, whereas men exhibited better general empathy than women.

Finally, our results revealed that students who prefer clinical specialties have better active listening and empathy skills than those who show preference for surgical specialties.

## Figures and Tables

**Table 1:** Sociodemographic characteristics of the sample.

		No.	%
Gender	Male	248	27
	Female	669	73
Civil status	Parents	395	58.3
	Shared flat	255	37.7
	Others	27	4
Parents working in health sciences	Yes	191	28.2
	No	486	71.8
Medical specialty	Clinical	318	59.3
	Surgical	147	27.4
	Laboratory	11	2.1
	Clinical-surgical	60	11.2

**Table 2:** Gender-based differences in active listening and empathy.

	Scales	Gender	No.	Mean	SD	Mann–Whitney U	*p*-Value
**Active listening**	Don’t interrupt	Men	189	3.33	1.139	46,604	0.371
		Women	515	3.25	1.122		
	100% attention	Men	189	2.95	1.136	46,947	0.457
		Women	515	2.86	1.162		
	Listen beyond words	Men	189	3.79	1.039	61,701	0.000 ***
		Women	515	4.29	0.796		
	Encourage to deeper	Men	189	4.18	0.905	54201.5	0.011 *
		Women	515	4.38	0.742		
**Empathy**	Adoption of perspectives	Men	248	71.39	24.774	81,659.5	0.821
		Women	665	69.54	26.912		
	Emotional compression	Men	248	78.13	24.369	82,270	0.929
		Women	666	76.26	26.004		
	Empathetic stress	Men	248	54.86	30.921	84,289	0.605
		Women	665	56.16	29.994		
	Empathetic joy	Men	248	63.76	28.289	87,905.5	0.133
		Women	666	67.15	27.626		
	General empathy	Men	248	72.70	26.243	74,473	0.024 *
		Women	665	68.89	25.575		

(SD = standard deviation) (*p < 0.05; ***p < 0.001).

**Table 3:** Scores in active listening and empathy of students on the basis of specialty preferences.

		Medical Specialty	No.	Mean	SD	Mann–Whitney U	*p*-Value
**Active listening**	Don´t interrupt	Clinical	238	3.29	1.134	12,603	0.562
		Surgical	110	3.21	1.084		
	100% attention	Clinical	238	2.79	1.112	12246	0.315
		Surgical	110	2.95	1.156		
	Listen beyond words	Clinical	238	4.19	0.860	12,518	0.480
		Surgical	110	4.19	1.027		
	Encourage to deeper	Clinical	238	4.32	0.806	11,359.5	0.031 *
		Surgical	110	4.14	0.840		
**Empathy**	Adoption of perspectives	Clinical	318	70.33	25.629	22,310.5	0.499
		Surgical	146	71.08	26.913		
	Emotional compression	Clinical	318	75.14	26.491	22,192.5	0.445
		Surgical	146	77.55	24.394		
	Empathetic stress	Clinical	318	57.55	29.012	20,420	0.048 *
		Surgical	145	51.39	32.217		
	Empathetic joy	Clinical	318	68.25	26.944	21,971	0.353
		Surgical	146	64.60	29.914		
	General empathy	Clinical	318	70.95	24.802	22,957.5	0.206
		Surgical	146	69.38	27.649		

(SD = standard deviation) (*p < 0.05).
